# *Gilvimarinus xylanilyticus* sp. nov., a novel 1,3-xylanase-secreting bacterium isolated from a marine green alga

**DOI:** 10.3389/fmicb.2022.1006116

**Published:** 2022-10-24

**Authors:** Yan-Jiao Zhang, Hai-Ning Sun, Ting-Ting Xu, Dian-Li Zhao, Chun-Mei Yu, Yi Zhang, Xi-Ying Zhang, Xiu-Lan Chen, Yu-Qiang Zhang, Fang Zhao

**Affiliations:** ^1^Shandong Province Key Laboratory of Applied Mycology, College of Life Sciences, Qingdao Agricultural University, Qingdao, China; ^2^State Key Laboratory of Microbial Technology, Marine Biotechnology Research Center, Shandong University, Qingdao, China; ^3^College of Marine Life Sciences & Frontiers Science Center for Deep Ocean Multispheres and Earth System, Ocean University of China, Qingdao, China; ^4^Laboratory for Marine Biology and Biotechnology, Pilot National Laboratory for Marine Science and Technology (Qingdao), Qingdao, China

**Keywords:** *Gilvimarinus*, polyphasic taxonomy, sp. nov., 1,3-xylanase-secreting bacteria, 1,3-xylanases

## Abstract

1,3-xylan, an important organic carbon in the ocean, is peculiar to marine algae. 1,3-xylanase-secreting bacteria and their extracellular 1,3-xylanases play pivotal roles in the degradation and biomass conversion of 1,3-xylan. However, only a few 1,3-xylanase-secreting bacteria and 1,3-xylanases have been reported. Here, we identified a novel marine bacterium capable of secreting 1,3-xylanases, designated as strain HB14^T^. Phylogenetic analysis revealed that strain HB14^T^ clustered tightly with known species of the genus *Gilvimarinus*, showing the highest 16S rRNA gene sequence similarity (97.7%) with the type strain of *Gilvimarinus chinensis*. Based on phylogenetic, genomic, chemotaxonomic and phenotypic studies, strain HB14^T^ was classified as a representative of a novel species in the genus *Gilvimarinus*, for which the name *Gilvimarinus xylanilyticus* sp. nov. was proposed. The type strain is HB14^T^ (=CCTCC AB 2022109^T^ = KCTC 92379^T^). Four 1,3-xylanases secreted by strain HB14^T^ were identified based on genome and secretome analyses, and the two (Xyn65 and Xyn80) with relatively higher abundance in secretome were successfully expressed in *Escherichia coli* and biochemically characterized. They showed the highest activity at pH 6.0–7.0 and 40°C and released mainly 1,3-xylobiose and 1,3-xylotriose from 1,3-xylan. These data suggest that strain HB14^T^ acts as a player in marine 1,3-xylan degradation and recycling and that its extracellular 1,3-xylanases may have a good potential in 1,3-xylooligosaccharides preparation.

## Introduction

The genus *Gilvimarinus*, belonging to the family *Cellvibrionaceae* in the class *Gammaproteobacteria*, was proposed by Du et al. (2009). Currently, four members have been reported in this genus, including *G. chinensis* (Du et al., 2009), ‘*G. agarilyticus*’ (Kim et al., 2011), *G. polysaccharolyticus* (Cheng et al., 2015) and *G. japonicus* (Kouzui et al., 2016). *Gilvimarinus* species are Gram-stain-negative, motile, and rod-shaped bacteria having diphosphatidylglycerol, phosphatidylglycerol and phosphatidylethanolamine as the major polar lipids and ubiquinone-8 as the major quinone (Cheng et al., 2015). All species in the genus *Gilvimarinus* have been isolated from coastal environments, including seawater, sea sand, seaweed and debris (Du et al., 2009; Kim et al., 2011; Cheng et al., 2015; Kouzui et al., 2016), where phytoplankton blooms frequently occur. Furthermore, type strains of all *Gilvimarinus* species were reported to possess the capability to degrade algal polysaccharides, especially agar and cellulose (Du et al., 2009; Kim et al., 2011; Cheng et al., 2015; Kouzui et al., 2016; Kouzuma and Fujii, 2018; Lee et al., 2018, 2021). Thus, they likely play important ecological roles in seaweed decomposition in coastal marine environments.

1,3-xylan is a homopolymer composed of β-1,3-linked D-xylopyranose units, which is peculiar to marine algae and is the main component in the cell walls of some red and green algae (Iriki et al., 1960; Veluraja and Atkins, 1987; Hsieh and Harris, 2019; Qeshmi et al., 2020). 1,3-xylan is considered the main xylan structure and an important organic carbon in the ocean (Qeshmi et al., 2020; Sun et al., 2021). In addition, 1,3-xylan-containing algal biomass serves as a potential feedstock for producing useful chemical commodities and functional 1,3-xylooligosaccharides (Goddard-Borger et al., 2012; Maeda et al., 2012; Umemoto et al., 2012). 1,3-xylanase-secreting bacteria and their extracellular 1,3-xylanases (EC 3.2.1.32) play pivotal roles in the degradation and biomass conversion of 1,3-xylan in the ocean. So far, some 1,3-xylanase-secreting bacteria and the 1,3-xylanases they secrete have been reported, including *Vibrio* (Araki et al., 2000; Kiyohara et al., 2005), *Pseudomonas* (Aoki and Kamei, 2006; Liang et al., 2015), *Alcaligenes* (Okazaki et al., 2002), *Thermotoga* (Okazaki et al., 2013) and *Flammeovirga* (Cai et al., 2018; Yi et al., 2020) species. 1,3-xylanases catalyze the hydrolysis of β-1,3-xylosidic linkages in 1,3-xylan. In the Carbohydrate Active Enzymes (CAZy) database^1^ (Lombard et al., 2014), all the reported 1,3-xylanases are classified in glycoside hydrolase (GH) family 26. Despite these, more 1,3-xylan-secreting marine bacteria and 1,3-xylanases are required to be identified and studied for a comprehensive understanding of the degradation and recycling of marine 1,3-xylan and for the exploration of 1,3-xylanases with good biotechnological/industrial potentials.

During an investigation of 1,3-xylan-secreting bacteria from green algal samples collected from a *Caulerpa lentillifera* aquaculture base in Wenchang, Hainan, China, a strain, designated HB14^T^, was isolated from a green alga (*Chaetomorpha* sp.; Sun et al., 2021). Strain HB14^T^ was prominent by its low 16S rRNA gene sequence similarity (97.7%) to known species in the genus *Gilvimarinus* and its ability to secrete 1,3-xylanases on the plate containing 1,3-xylan (Sun et al., 2021). In this study, the exact taxonomic position of strain HB14^T^ was determined by using a polyphasic approach. Moreover, four extracellular 1,3-xylanases secreted by strain HB14^T^ were identified by genome and secretome analyses, and two of these 1,3-xylanases were successfully expressed in *E. coli* and biochemically characterized. The results indicated that they may have a potential in the preparation of 1,3-xylooligosaccharides from 1,3-xylan.

## Materials and methods

### Cultivation of bacterial strains

Strain HB14^T^ was isolated from a green alga (*Chaetomorpha* sp.) collected from a *Caulerpa lentillifera* aquaculture base in Wenchang, Hainan, China (Sun et al., 2021). Strain *G. chinensis* CGMCC 1.7008^T^ was purchased from CGMCC and characterized alongside for comparative purposes. Unless otherwise stated, the strains were routinely cultivated in MBC [marine broth 2216 (MB; Difco) supplemented with 0.05% (w/v) cellobiose] or on MAC [marine broth 2216, 1.5% (w/v) agar and 0.05% (w/v) cellobiose] at 30°C.

### Phylogenetic analysis

The 16S rRNA gene of strain HB14^T^ was amplified by PCR with the universal primers 27F (5’-AGAGTTTGATCCTGGCTCAG-3′) and 1492R (5’-GGTTACCTTGTTACGACTT-3′; Weisburg et al., 1991). The amplification product was cloned into the pClone007 Versatile Simple Vector (Tsingke, China), transferred into *E. coli* DH5α (Vazyme, China) and further sequenced at Tsingke (Qingdao, China). The obtained 16S rRNA gene sequence was compared to those of species validly published through the EzBioCloud server^2^ (Yoon et al., 2017a). Phylogenetic trees were constructed with MEGA 11 (Tamura et al., 2021) using maximum-likelihood (Felsenstein, 1981), neighbor-joining (Saitou and Nei, 1987) and maximum-parsimony (Fitch, 1971) methods. The topologies of the phylogenetic trees were evaluated by bootstrap analyses (1,000 replications).

### Genomic analysis

Genomic DNA of strain HB14^T^ was extracted and sequenced at Biozeron (Shanghai, China) using a bacterial DNA Kit (Omega, United States) and the Illumina NovaSeq 6,000, respectively. The DNA G + C % content was calculated directly from the draft genome sequence. The obtained draft genome was annotated using the NCBI Prokaryotic Genome Annotation Pipeline (PGAP; Tatusova et al., 2016) and RAST annotation server (Brettin et al., 2015). The digital DNA–DNA hybridization (dDDH) value and the average nucleotide identity (ANI) were calculated using the genome-to-genome distance calculator (GGDC 2.1) online service^3^ (Meier-Kolthoff et al., 2013) and the ChunLab’s online ANI calculator^4^ (Yoon et al., 2017b), respectively. The average amino acid identity (AAI) was calculated using an AAI calculator server^5^ (Rodríguez-R and Konstantinidis, 2014). The percentage of conserved proteins (POCP) was estimated using an approach described by Qin et al. (Qin et al., 2014).

### Chemotaxonomic analysis

For the analysis of cellular fatty acids, cells were cultivated in MBC at 30°C and collected at the exponential phase. Fatty acid methyl esters were analyzed by gas chromatograph (Agilent 6,850 N) and identified using the Sherlock Microbial Identification System (MIDI, version 6.1) at Yellow Sea Fisheries Research Institute, Chinese Academy of Fishery Sciences (Qingdao, China). For the analysis of polar lipids and quinone, bacterial cells were cultivated in MBC at 30°C and collected at the early stationary phase. Polar lipids were extracted according to the method described by Komagata and Suzuki (Komagata and Suzuki, 1988) and analyzed using the two-dimensional thin layer chromatography (TLC) on silica gel 60 F254 plates (Merck, Germany) with appropriate spraying reagents including ethanolic molybdophosphoric acid (total lipids), ninhydrin (aminolipids) and molybdenum blue (phospholipids). The respiratory quinone of strain HB14^T^ was extracted, separated and analyzed with the method described by Li et al. (Li et al., 2021a).

### Morphological, physiological, and biochemical characterization

Cellular morphology was observed by transmission electron microscope (TEM, FEI Tecnai G2 F20) with strain HB14^T^ cells from exponentially growing cultures. Colony morphology was observed after incubation on MAC at 30°C for 4 days. Gram staining and agar corrosion were carried out according to the method described by Tindall et al. (Tindall et al., 2007). Growth at different temperatures (4, 10, 15, 20, 25, 30, 37, 45 and 50°C) was determined in MBC. Growth at different NaCl concentrations [0, 1.0–15.0% (at an interval of 2.0% units), 16 and 17%; w/v] was determined in NP broth (Zhang et al., 2017) supplemented with 0.05% (w/v) cellobiose at 30°C. Growth tests for pH range [pH 5.0–10.0, at an interval of 0.5 pH units, buffered with MES (pH 5.0–6.0, 50 mM), MOPS (pH 6.5–7.0, 50 mM), Tris (pH 7.5–9.0, 50 mM) and CHES (pH 9.5–10.0, 50 mM)] were performed in NP broth supplemented with 0.05% (w/v) cellobiose and 3.0% (w/v) NaCl. Oxidase activity was tested by using commercial oxidase test strips (Tianhe Microorganism Reagent, China) according to the manufacturer’s instructions. Catalase activity was determined by bubble production in 3.0% (v/v) H_2_O_2_. Hydrolysis of casein (1.0%, w/v), starch (0.2%, w/v) and Tween 80 (1.0%, v/v) was examined by using MAC as the basal medium and incubation at 30°C. Cellulase activity was tested according to the method described by Kasana et al. (Kasana et al., 2008). Growth under the anaerobic condition was determined in MBC supplemented with potassium nitrate (0.1%, w/v), cysteine hydrochloride (0.05%, w/v) and sodium sulfide (0.05%, w/v) in Hungate tubes filled with oxygen-free N_2_ at 30°C for 2 weeks. Susceptibility to antibiotics was examined by the disk-diffusion method on MAC. More biochemical characteristics were determined using API ZYM and API 20NE strips (BioMérieux, France) following the manufacturers’ instructions with sea salts (3.0%, w/v, Sigma, United States) for cell suspension. Carbon utilization was determined using Biolog GEN III (Biolog, United States) according to the manufacturer’s instructions with inoculation fluid B for cell suspension.

### Growth experiment and extracellular 1,3-xylanase assay

1,3-xylan was prepared as previously described (Sun et al., 2021). Growth of strain HB14^T^ on 1,3-xylan was determined at 30°C and pH 7.5 with 0.2% (w/v) 1,3-xylan as the sole carbon source by using 0.2% (w/v) xylose and glucose as the control substrates. The compositions of the medium used were as previously described (Sun et al., 2021). During cultivation, the optical density (OD) at 600 nm of the cultures was measured to generate growth curves.

During the cultivation of strain HB14^T^ on 1,3-xylan, the cultures (1 ml) taken at an interval of 12 h were centrifuged (12,857 g at 4°C for 5 min), and the supernatants were collected for determining the extracellular 1,3-xylanase activity. To compare the extracellular 1,3-xylanase activities induced by 1,3-xylan, xylose and glucose, culture supernatants on each substrate at 84 h were collected and the extracellular 1,3-xylanase activities were determined separately. The extracellular 1,3-xylanase activity was measured by the dinitrosalicylic acid (DNS) method (Miller et al., 1960). The reaction system contained 10 μl culture supernatant and 90 μl 1,3-xylan (1.0%, w/v) in the phosphate-buffered saline (PBS, 20 mM, pH 7.0) and incubated at 30°C for 3.5 h. The reaction was terminated by the addition of 200 μl DNS. Then the reaction mixture was boiled at 100°C for 5 min and the OD_550 nm_ was measured to quantify the released xylose. One unit of enzyme activity (1 U) is defined as the amount of enzyme required to release 1 μmol xylose per min.

### Bioinformatic analysis of 1,3-xylanases

The putative 1,3-xylanases of strain HB14^T^ were predicted according to genomic annotations and dbCAN analyses (Yin et al., 2012). The signal peptides of 1,3-xylanases were predicted by using SignalP 5.0 (Armenteros et al., 2019). Domain analysis was performed in the NCBI conserved domain database (CDD; Marchler-Bauer et al., 2017) and the Pfam database (Mistry et al., 2021). The molecular weights of 1,3-xylanases were calculated by ExPASy.^6^ Multiple amino acid sequence alignment was carried out with CLC Sequence Viewer 6 and ESPript 3.0 (Robert and Gouet, 2014).

### Secretome analysis

Strain HB14^T^ was cultivated on 1,3-xylan at 30°C for 60 h, and then 20 ml of the culture was collected and centrifuged (12,857 g for 10 min at 4°C). The supernatant was moved into an ultrafiltration tube (molecular weight cut-off of 3 kDa) and centrifuged (4,476 g at 4°C) for concentration. Then, the concentrated sample (300 μl) was sent to Applied Protein Technology (Shanghai, China) for secretome analysis. In brief, the sample was reduced by dithiothreitol, alkylated by iodoacetamide and digested by trypsin. Then, peptides in the sample were desalted for liquid chromatography-mass spectroscopy/mass spectroscopy (LC–MS/MS) analysis. Finally, the raw data was analyzed by Thermo Scientific Proteome Discoverer™ 1.4 with the genomic data of strain HB14^T^ as the database. The secretome data has been uploaded to the ProteomeXchange Consortium via the PRIDE under the accession number PXD035564.

### Gene cloning, enzyme expression and purification

The genes *xyn65* and *xyn80* without the signal peptide were cloned from the genomic DNA of strain HB14^T^ by PCR amplification and inserted into the pET22b vector (Novagen, Germany). The primers used are shown in Supplementary Table S1. The constructed plasmids were transferred into *E. coli* BL21(DE3; Vazyme, China). The cells were cultivated in the Luria-Bertani (LB) medium containing 0.1 mg/ml ampicillin at 37°C and 180 rpm to OD_600_ ≈ 1.0 and then induced at 18°C and 120 rpm for 18 h with 0.5 mM isopropyl-β-D-thiogalactopyranoside (IPTG). The cells were harvested by centrifugation (11,500 g for 5 min at 4°C) and lysed in a lysis buffer [50 mM Tris–HCl, 100 mM NaCl, 0.5% (v/v) glycerol, pH 8.0] by a pressure crusher (JNBIO JN-02C). The recombinant proteins were purified by the nickel-nitrilotriacetic acid resin (GE Healthcare, United States) and then desalted by PD-10 columns (GE Healthcare, United States). Protein purity and apparent molecular weight were determined by SDS-PAGE. Protein concentration was determined by the bicinchoninic acid (BCA) method using a BCA protein assay kit (Thermo, United States).

### Biochemical characterization of recombinant 1,3-xylanases

The enzyme assays of Xyn65 and Xyn80 on xylans were determined by using the DNS method (*vide supra*). The standard reaction mixture contained 10 μl enzyme solution (36.8 μM for Xyn65 or 31.4 μM for Xyn80) and 90 μl xylan (1.0%, w/v) in PBS (20 mM, pH 7.0) and was incubated at 40°C for 10 min.

The substrate specificities of Xyn65 and Xyn80 were determined at 40°C in PBS (20 mM, pH 7.0) with 1,3-xylan, wheat arabinoxylan (Megazyme, Ireland), beechwood xylan (Megazyme, Ireland) and 1,3/1,4-xylan (Elicityl, France) as substrates at a concentration of 1.0% (w/v). The effect of temperature on enzyme activities was determined with 1,3-xylan as substrate in PBS (20 mM, pH 7.0) from 10°C to 60°C at an interval of 10°C. The effect of pH on enzyme activities was determined with 1,3-xylan as substrate at 40°C in the Britton-Robinson buffer from pH 3.0 to 10.0 at an interval of 1.0 pH units.

### Analysis of the released products from 1,3-xylan by recombinant 1,3-xylanases

The reaction mixture containing 20 μl enzyme solution (36.8 μM for Xyn65 or 31.4 μM for Xyn80) and 180 μl 1,3-xylan (1.0%, w/v) in PBS (20 mM, pH 7.0) was incubated at 40°C. At 0 h, 1 h and 24 h of the reaction, aliquots (200 μl) were taken out, boiled for 5 min and analyzed by gel filtration on a Superdex 30 Increase 10/300 GL column (GE Healthcare, United States) using high-performance liquid chromatography (Shimadzu LC-20AT) equipped with an evaporative light scattering detector (Shimadzu ELSD LT II; HPLC-ELSD). The injected volume was 20 μl. The products were eluted with deionized water for 65 min with a flow rate of 0.3 ml/min. A mixture of xylose, 1,3-xylobiose and 1,3-xylotriose (5 mM each) was used as the external standard. 1,4-xylohexaose (5 mM) was used as the internal standard. 1,3-xylobiose and 1,3-xylotriose were prepared from 1,3-xylan hydrolyzed by 1,3-xylanase Xyl4 as described previously (Sun et al., 2021).

## Results and discussion

### Phylogenetic and genomic analyses of strain HB14^T^

**Figure 1:**
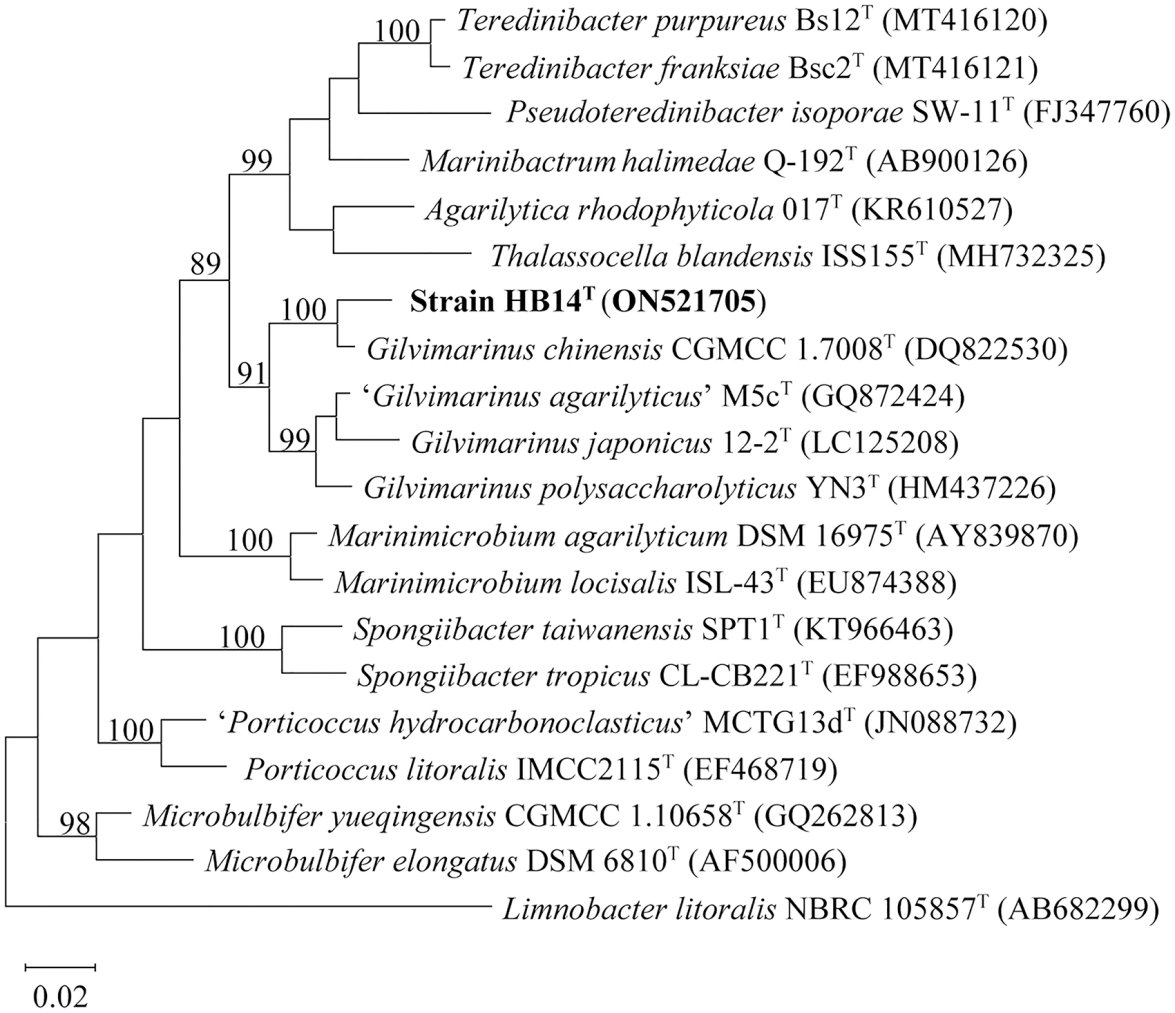
Maximum-likelihood phylogenetic tree based on 16S rRNA gene sequences of strain HB14^T^ (in bold) and the type strains of its closely related species. Bootstrap values (>70%) based on 1,000 replicates are shown at the branching points. Bar, 0.02 substitutions per nucleotide position. *Limnobacter litoralis* NBRC 105857^T^ was used as the outgroup.

Strain HB14^T^ was isolated from the surface of a marine green alga (*Chaetomorpha* sp.; Sun et al., 2021). The nearly full-length 16S rRNA gene sequence (1,496 bp, ON521705) of strain HB14^T^ was obtained by PCR, which was identical to that extracted from its genomic sequence. HB14^T^ showed the highest 16S rRNA gene sequence similarity to the type strain of *G. chinensis* (97.7%), followed by those of *G. polysaccharolyticus* (94.8%) and *G. japonicus* (93.7%), and less than 93.0% to the type strains of other species in the family *Cellvibrionaceae*, which were all below the threshold (98.7%) for bacterial species delineation (Kim et al., 2014). In the maximum-likelihood tree based on the 16S rRNA gene sequences, strain HB14^T^ fell within the clade comprising known members of the genus *Gilvimarinus* (Figure 1) with high bootstrap support (91%), where it formed a well-supported (bootstrap value of 100%) peripheral branch together with *G. chinensis*. This stable topology was also supported by the neighbor-joining (Supplementary Figure S1) and maximum-parsimony trees (Supplementary Figure S2). These results suggested that strain HB14^T^ belongs to the genus *Gilvimarinus* and represents a novel species within the genus.

The assembled draft genome of strain HB14^T^ (JAMFTH000000000) was 3,930,865 bp in length, with 13 contigs and an N50 value of 2,053,438 bp. According to the annotation by PGAP, a total of 3,427 genes were predicted, including 3,361 protein-coding genes, 49 RNA genes and 17 pseudogenes. The calculated genomic DNA G + C content of strain HB14^T^ was 54.0 mol%, a value higher than that of *G. chinensis* CGMCC 1.7008^T^ (51.2 mol%), the closest relative (Du et al., 2009). Further, the ANI and dDDH values between the genomes of strain HB14^T^ and *G. chinensis* CGMCC 1.7008^T^ were 77.1 and 21.8%, respectively, which were all significantly lower than the suggested cut-off values for ANI (95.0–96.0%) and dDDH (70.0%) used to discriminate bacterial species (Goris et al., 2007; Richter and Rosselló-Móra, 2009; Meier-Kolthoff et al., 2013). The AAI and POCP values between the genomes of strain HB14^T^ and *G. chinensi*s CGMCC 1.7008^T^ were 78.9 and 77.9%, respectively, which were higher than the suggested cut-off values for AAI (76.0%) and POCP (50%) to delimit bacterial genera (Qin et al., 2014; Nicholson et al., 2020; Li et al., 2021b). Altogether, the genomic analyses indicated that strain HB14^T^ represents a novel species of the genus *Gilvimarinus*, supporting our conclusion derived from the phylogenetic analysis based on 16S rRNA gene sequences.

### Chemotaxonomic characteristics

The predominant cellular fatty acids (>10.0%) of strain HB14^T^ were summed feature 3 (contains C_16:1_ *ω*6*c* and/or C_16:1_ *ω*7*c*), summed feature 8 (contains C_18:1_ *ω*7*c* and/or C_18:1_ *ω*6*c*) and C_16:0_, similar to those of *G. chinensis* CGMCC 1.7008^T^ (Supplementary Table S2). The main polar lipids of strain HB14^T^ were diphosphatidylglycerol, phosphatidylglycerol and phosphatidylethanolamine (Supplementary Figure S3). The predominant respiratory quinone of strain HB14^T^ was ubiquinone-8, common for members of the family *Cellvibrionaceae*.

### Morphological, physiological, and biochemical characteristics

**Figure 2:**
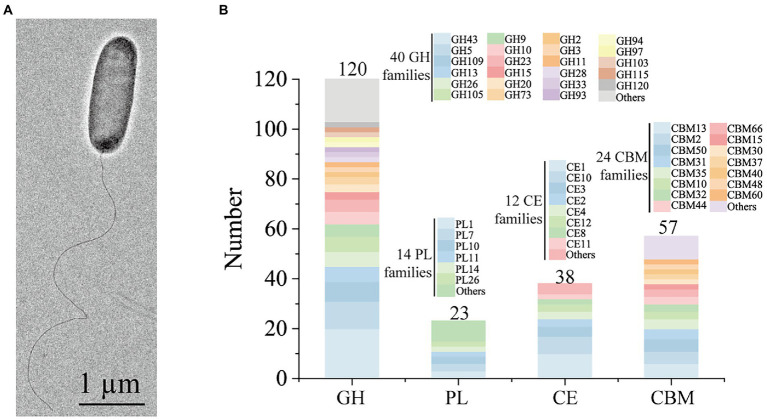
Morphological and genomic analysis of strain HB14^T^ possessing strong polysaccharide degradation capabilities. **(A)** Cell morphological observation of strain HB14^T^ by transmission electron micrograph. Strain HB14^T^ cells for observation were incubated on MAC at 30°C for 3 days. **(B)** Quantification of the carbohydrate-active enzymes (CAZymes) and their family classifications encoded by the genome of strain HB14^T^. GH, glycoside hydrolase; PL, polysaccharide lyase; CE, carbohydrate esterase; CBM, carbohydrate-binding module.

Cells of strain HB14^T^ were Gram-stain-negative, rod-shaped and motile with a single polar flagellum (Figure 2A). Strain HB14^T^ was resistant to cephalexin (30 μg), but sensitive to ampicillin (10 μg), carbenicillin (100 μg), chloramphenicol (30 μg), colistin sulphate (10 μg), erythromycin (15 μg), gentamicin (10 μg), neomycin (10 μg), novobiocin (5 μg), oleandomycin (15 μg), penicillin G (10 units), polymyxin B (300 units), streptomycin (10 μg), tetracycline (30 μg) and vancomycin (30 μg). The differential characteristics between strain HB14^T^ and *G. chinensis* CGMCC 1.7008^T^ were listed in Table 1. More detailed morphological, physiological and biochemical characteristics of strain HB14^T^ were presented in the species description.

**Table 1:** Differential characteristics of strain HB14^T^ and *G. chinensis* CGMCC 1.7008^T^.

Characteristic	1	2
*Cell size (μm)*		
Width	0.3–0.7	0.6–0.7
Length	1.1–2.7	1.5–2.5
*Growth with/at*		
4°C	−	+
15.0% (w/v) NaCl	+	−
*Hydrolysis of*		
Agar	−	+
Urea	+	−
Nitrate reduction	+	−
Acid production from glucose	−	w
*Enzyme activity (API ZYM)*		
Esterase lipase (C8)	+	−
β-galactosidase	+	−
G + C content (mol%)	54.0	51.2
Isolation source	Marine alga	Coastal seawater

Strains: 1, HB14^T^; 2, *G. chinensis* CGMCC 1.7008^T^. All data are from this study, except for morphological properties and the DNA G + C content of *G. chinensis* CGMCC 1.7008^T^, which are from Du et al. (2009). +, positive; −, negative; w, weakly positive.

### The ability of strain HB14^T^ to utilize 1,3-xylan and secrete 1,3-xylanases

**Figure 3:**
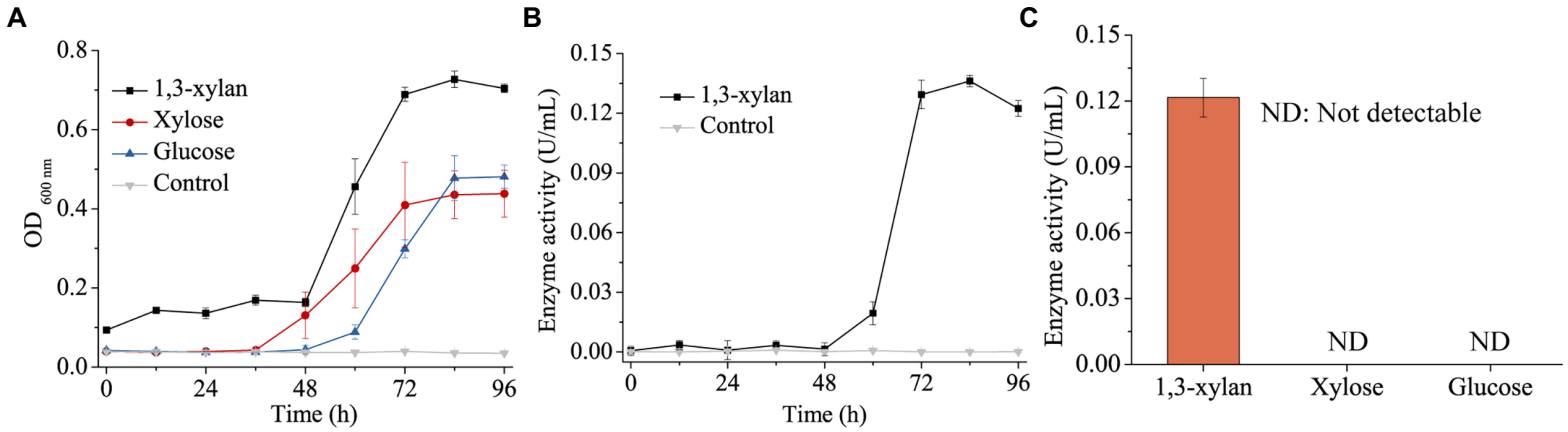
Growth of strain HB14^T^ on 1,3-xylan and the induction of extracellular 1,3-xylanase activity. **(A)** Growth curves of strain HB14^T^ on 1,3-xylan, xylose and glucose. Strain HB14^T^ was cultivated at 30°C and pH 7.5 with 0.2% (w/v) 1,3-xylan, xylose or glucose as the sole carbon source. The culture without carbon source was treated as the control. **(B)** The extracellular 1,3-xylanase activities during the cultivation of strain HB14^T^ on 1,3-xylan. The 1,3-xylanase activities were determined at 30°C in PBS (pH 7.0). The extracellular 1,3-xylanase activity of strain HB14^T^ cultivated without carbon source was treated as the control. **(C)**, The extracellular 1,3-xylanase activity of strain HB14^T^ grown on 1,3-xylan, xylose or glucose at the late-log phase. Cultures of strain HB14^T^ at 84 h on each substrate of 1,3-xylan, xylose and glucose were collected separately. Then, the extracellular 1,3-xylanase activities of the culture were determined at 30°C in PBS (pH 7.0). The data shown in the graph are from triplicate experiments (mean ± S.D.).

Some *Gilvimarinus* strains have been reported to exhibit the capability to degrade marine algal polysaccharides, such as agar, cellulose, carrageenan and laminarin (Du et al., 2009; Kim et al., 2011; Cheng et al., 2015; Kouzui et al., 2016; Kouzuma and Fujii, 2018; Lee et al., 2018, 2021). Based on the genomic annotation and dbCAN analysis, the genome of strain HB14^T^ encodes 120 GHs, 23 polysaccharide lyases (PL), 38 carbohydrate esterases (CE) and 57 carbohydrate-binding modules (CBM), distributed in 40, 14, 12 and 24 families, respectively (Figure 2B). These data suggested the potential capability of strain HB14^T^ to degrade polysaccharides. Strain HB14^T^ has been shown to form an apparent hydrolytic zone on the plate containing 1,3-xylan (Sun et al., 2021), suggesting that it can secrete 1,3-xylanase. To further investigate its 1,3-xylan-utilizing ability and extracellular 1,3-xylanase activity, the growth of strain HB14^T^ was determined in a liquid medium with 1,3-xylan as the sole carbon source, with xylose and glucose as the control carbon sources. Strain HB14^T^ was able to grow on 1,3-xylan, as well as xylose and glucose (Figure 3A), indicating that it could utilize 1,3-xylan for growth. In the culture supernatant of strain HB14^T^ grown on 1,3-xylan, the 1,3-xylanase activity reached the highest level (0.14 ± 0.003 U/ml) at the late-log phase (Figure 3B). In contrast, no extracellular 1,3-xylanase activity was detected when strain HB14^T^ was grown on xylose or glucose (Figure 3C), suggesting that the extracellular 1,3-xylanases were 1,3-xylan inducible.

### Identification of the 1,3-xylanases secreted by strain HB14^T^

**Figure 4:**
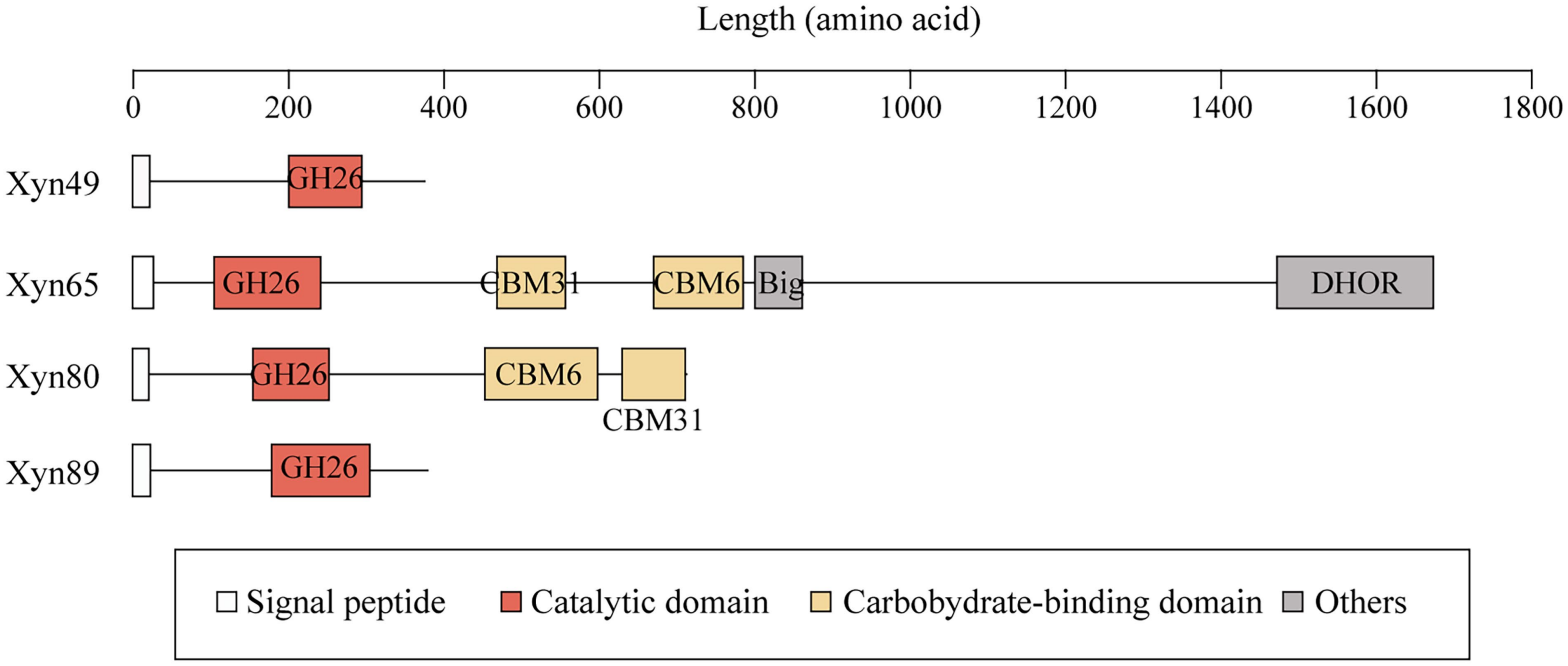
The domain architectures of the four 1,3-xylanases secreted by strain HB14^T^. Signal peptides were predicted by SignalP 5.0. Domain analysis was performed in the NCBI CDD database and the Pfam database. Domains were illustrated in different colors based on their functional annotations. Big, bacterial immunoglobulin (Ig)-like domain; DHOR, di-haem oxidoreductase superfamily.

The genome of strain HB14^T^ encodes four putative 1,3-xylanases, which were named Xyn49, Xyn65, Xyn80 and Xyn89 (Table 2). All the four 1,3-xylanases have a signal peptide predicted by SignalP 5.0 (Figure 4), implying that they are likely secretory enzymes. Based on secretome analysis, the four putative 1,3-xylanases were all identified in the culture of strain HB14^T^ cultivated with 1,3-xylan as the sole carbon source. Of these, Xyn65 was the most abundant (82.3%), followed by Xyn80 (12.0%), Xyn49 (4.5%) and Xyn89 (1.2%; Table 2). The results suggested that these 1,3-xylanases were all likely involved in the extracellular degradation of 1,3-xylan of strain HB14^T^, and that Xyn65 might be the most important one.

**Table 2:** Information of the extracellular 1,3-xylanases secreted by strain HB14^T^ identified by secretome analysis.

1,3-xylanase	Accession number	Top identity with (Identity)^a^	Molecular weight (kDa)	PSMs^b^	Abundance (%)^c^
Xyn49	WP_253968357.1	Xyl4 (33.7%)	40.0	15	4.5
Xyn65	WP_253968372.1	Xyl4 (56.0%)	176.6	275	82.3
Xyn80	WP_253969254.1	Xyl4 (30.0%)	74.8	40	12.0
Xyn89	WP_253969263.1	Xyl4 (31.3%)	40.0	4	1.2

a The amino acid sequence identities were obtained by BLASTp against the UniProtKB database in NCBI with the catalytic domains of the four 1,3-xylanases.

b PSMs, peptide-spectrum matches.

c Abundance was calculated based on the proportion of the PSMs of the 1,3-xylanase in the sum of PSMs of all 1,3-xylanases in the secretome.

Several 1,3-xylanases have been reported, which are derived from marine *Vibrio* (Araki et al., 2000; Kiyohara et al., 2005), *Alcaligenes* (Okazaki et al., 2002), *Pseudomonas* (Aoki and Kamei, 2006; Liang et al., 2015), *Thermotoga* (Okazaki et al., 2013) and *Flammeovirga* (Cai et al., 2018; Yi et al., 2020). They are generally modular proteins containing one GH26 catalytic domain and one or more CBMs, mainly belonging to CBM6 and CBM31 (Table 3). The function of the CBMs is to bind insoluble 1,3-xylan and facilitate the hydrolysis of 1,3-xylan (Okazaki et al., 2002). In addition to modular members, Xyn26A and Xyl512 are two single-domain 1,3-xylanases, consisting of only one GH26 catalytic domain (Okazaki et al., 2013; Cai et al., 2018). Among the extracellular 1,3-xylanases of strain HB14^T^, according to domain architecture prediction, Xyn49 and Xyn89 are single-domain enzymes, and Xyn65 and Xyn80 are modular enzymes (Figure 4). They all have a GH26 catalytic domain (Figure 4), which exhibits top amino acid sequence identities (30.0–56.0%) with the previously reported 1,3-xylanase Xyl4 (Kiyohara et al., 2005; Table 2). The catalytic domain of Xyl4 have been structurally investigated with two glutamate residues as the catalytic residues (Goddard-Borger et al., 2012). Multiple sequence alignment indicated that the two residues are conserved in these four 1,3-xylanases secreted by strain HB14^T^ (Supplementary Figure S4), suggesting a similar catalysis mechanism. In addition to the GH26 domain, Xyn65 and Xyn80 also possess a CBM31 and a CBM6 (Figure 4). Moreover, Xyn65 also contains a bacterial immunoglobulin (Ig)-like domain (Big) and a domain belonging to the di-haem oxidoreductase (DHOR) superfamily. To our knowledge, the domain architecture of Xyn65 is distinct from any previously reported 1,3-xylanase (Figure 4). Big domains are commonly distributed in GHs, and may have different functions, such as binding carbohydrates (Xing et al., 2022), stabilizing the catalytic domain (Liu et al., 2010) and serving as a thermostabilizing module (da Silva et al., 2020). DHOR domains appear to act as di-heme oxidoreductases with probable peroxidase activity (Goonesekere et al., 2010), which, however, have not been found in GHs. We hypothesize that in Xyn65 (1) the DHOR domain may enable Xyn65 a novel activity (e.g., oxidoreductase activity) on 1,3-xylan; (2) although DHOR shows sequence similarities to di-heme oxidoreductases, it may evolve new functions (e.g., binding 1,3-xylan or stabilizing Xyn65 structure). Thus, Xyn65 is likely a new GH26 member with a novel structure, which awaits further investigation.

**Table 3:** Characteristics of Xyn65, Xyn80 and reported 1,3-xylanases.

Enzyme	Source bacteria	Domain architecture^a^	Specific activity (U/μmol)^b^	Optimum pH	Optimum temperature (°C)	Main products^c^	Reference
Xyn65	Strain HB14^T^	GH26-CBM31-CBM6-Big-DHOR	76.9 ± 4.7	6.0–7.0	40	DP2/3	This study
Xyn80	Strain HB14^T^	GH26-CBM6-CBM31	91.9 ± 0.6	6.0–7.0	40	DP2/3	This study
*Vi*TxyA	*Vibrio* sp. XY-214	GH26-CBM31	508.1	7.0	37	DP2/3	Araki et al. (2000); Araki et al. (1999)
*Al*TxyA	*Alcaligenes* sp. XY-234	GH26-CBM31	--	7.0	40	DP2/3	Okazaki et al. (2002)
Xyl4	*Vibrio* sp. AX-4	GH26-CBM31-CBM31	294.2	7.0–7.5	37	DP2/3/4	Kiyohara et al. (2005)
AxnB	*Pseudomonas* sp. ND137	GH26-CBM31-CBM6	--	--	--	--	Aoki and Kamei (2006)
Xyn26A	*Thermotoga* sp. DSM 4359	GH26	4,933.2	6.5	85	DP2/3	Okazaki et al. (2013)
XylII	*Pseudomonas* sp. MA103	GH26-CBM31-CBM31	1,263.4	7.5	35	DP1/2/3	Liang et al. (2015)
Xyl512	*Flammeovirga* sp. WPAGA1	GH26	86.7	7.0	20	DP1/2	Cai et al. (2018)
Xyl88	*Flammeovirga pacifica* WPAGA1	GH26-CBM (novel family)	--	7.0–8.0	45	DP1/2	Yi et al. (2020)

a GH, glycoside hydrolase; CBM, carbohydrate-binding module; Big, bacterial immunoglobulin (Ig)-like domain; DHOR, di-haem oxidoreductase superfamily.

b One unit of enzyme activity (U) is the amount of enzyme that liberates 1 μmol xylose from 1,3-xylan per min.

c DP, degree of polymerization.

### Expression and characterization of the 1,3-xylanases

**Figure 5:**
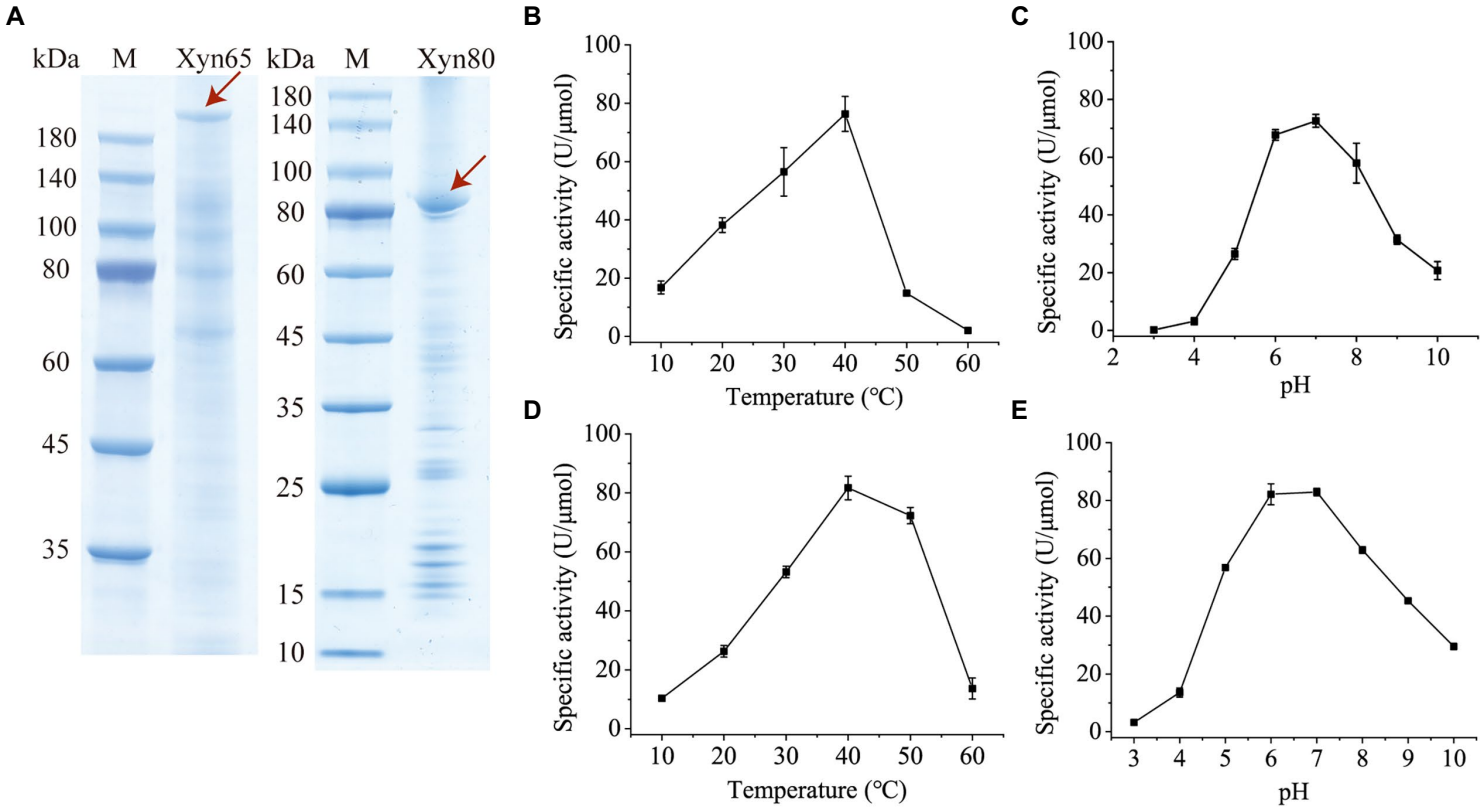
SDS-PAGE analysis and biochemical characterization of recombinant 1,3-xylanases Xyn65 and Xyn80. **(A)** SDS-PAGE analysis of Xyn65 and Xyn80. The enzyme bands are indicated by red arrows. M, protein molecular weight marker. **(B)** Effect of temperature on Xyn65 activity. **(C)** Effect of pH on Xyn65 activity. **(D)** Effect of temperature on Xyn80 activity. **(E)**, Effect of pH on Xyn80 activity. In B and D, the 1,3-xylanase activities were determined in PBS (pH 7.0) from 10°C to 60°C. In C and E, the 1,3-xylanase activities were determined in the Britton-Robinson buffer with different pH values (pH 3.0–10.0). The data shown in the graph are from triplicate experiments (mean ± S.D.).

**Figure 6:**
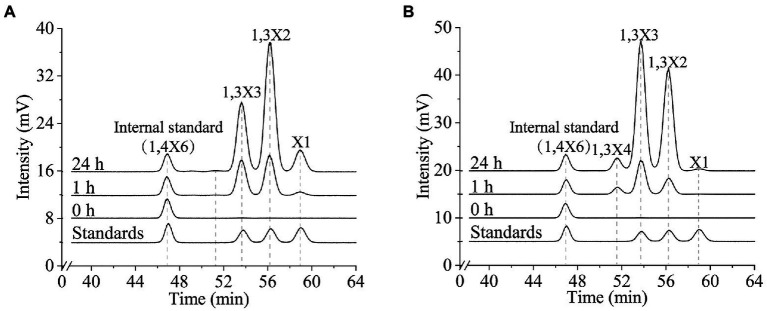
Time-course analysis of the 1,3-xylan degradation by recombinant 1,3-xylanases Xyn65 **(A)** and Xyn80 **(B)**. Xyn65 and Xyn80 were separately incubated with 1,3-xylan at 40°C in PBS (pH 7.0). At 0 h, 1 h and 24 h, the reaction was terminated by boiling for 5 min and the products were analyzed by HPLC-ELSD. A mixture of xylose, 1,3-xylobiose (1,3X2) and 1,3-xylotriose (1,3X3) was used as the external standard. 1,4-xylohexaose (1,4X6) was used as the internal standard. The data are representatives of the results of triplicate experiments.

To characterize the 1,3-xylanases secreted by strain HB14^T^, we attempted to express Xyn49, Xyn65, Xyn80 and Xyn89 in *E*. *coli*, but this was only successful in Xyn65 and Xyn80. SDS-PAGE analysis showed that the purified recombinant Xyn65 and Xyn80 exhibited apparent molecular weights of approximately 180 kDa and 80 kDa, respectively (Figure 5A), consistent with their theoretical molecular weights (Table 2). Substrate specificity analysis showed that Xyn65 and Xyn80 hydrolyzed only 1,3-xylan, with specific activities of 76.9 ± 4.7 U/μmol and 91.9 ± 0.6 U/μmol, respectively (Table 4), indicating that they are strict 1,3-xylanases. Both 1,3-xylanases displayed the highest activity at 40°C and pH 6.0–7.0 (Figures 5B–E). During the hydrolysis of 1,3-xylan by Xyn65 and Xyn80, 1,3-xylooligosaccharides with degrees of polymerization (DP) from one to four were detected, with 1,3-xylobiose and 1,3-xylotriose as the major final products (Figure 6), suggesting the endo-action pattern of both enzymes on 1,3-xylan. Together, Xyn65 and Xyn80 are two strict endo-1,3-xylanases with similar enzymatic characteristics.

**Table 4:** The substrate specificities of recombinant 1,3-xylanases Xyn65 and Xyn80.

Substrate	Specific activity (U/μmol)^a^	
	Xyn65	Xyn80
1,3-xylan	76.9 ± 4.7	91.9 ± 0.6
Wheat arabinoxylan	ND	ND
Beechwood xylan	ND	ND
1,3/1,4-xylan	ND	ND

a The substrate specificities were determined at 40°C in PBS (pH 7.0). The data shown in the graph are from triplicate experiments (mean ± S.D.). ND, no detectable enzyme activity.

All previously reported 1,3-xylanases are neutral enzymes, displaying the highest activity at pH 6.5–8.0 (Araki et al., 2000; Okazaki et al., 2002; Kiyohara et al., 2005; Aoki and Kamei, 2006; Okazaki et al., 2013; Liang et al., 2015; Cai et al., 2018; Yi et al., 2020). Except the cold-adapted Xyl512 (Cai et al., 2018) and the thermostable Xyn26A (Okazaki et al., 2013), the other 1,3-xylanases are all mesophilic enzymes. Their major products released from 1,3-xylan are generally xylose, 1,3-xylobiose, 1,3-xylotriose and 1,3-xylotetraose (Table 3). Similarly, Xyn65 and Xyn80 function at neutral pH and mesophilic conditions, releasing mainly 1,3-xylobiose and 1,3-xylotriose from 1,3-xylan. 1,3-xylanase is an effective tool in the conversion of 1,3-xylan and the produced 1,3-xylooligosaccharides could be the important ingredients in some functional foods (Cai et al., 2018). Although the specific activities of Xyn65 (76.9 ± 4.7 U/μmol) and Xyn80 (91.9 ± 0.6 U/μmol) are not very high compared with those of the reported 1,3-xylanases (86.7 ~ 4,933.2 U/μmol; Table 3), both enzymes released mainly 1,3-xylooligosaccharides from 1,3-xylan with a little xylose (Figure 6). They are two newly discovered candidates for the conversion of 1,3-xylan into 1,3-xylooligosaccharides, the potentials of which need further exploration.

## Conclusion

In this study, a taxonomic study was carried out on a 1,3-xylanase-secreting bacterium, strain HB14^T^, isolated from a marine green alga. Phylogenetic and genomic analyses indicated that strain HB14^T^ should be assigned to the genus *Gilvimarinus*. Strain HB14^T^ exhibits the typical characteristics of the genus *Gilvimarinus*, such as Gram-stain-negative, rod-shaped, oxidase- and catalase-positive, motile with a single polar flagellum, having ubiquinone-8 as the sole respiratory quinone, and having diphosphatidylglycerol, phosphatidylglycerol and phosphatidylethanolamine as the major polar lipids. However, strain HB14^T^ can be distinguished from its most closely related species *G. chinensis* by several phenotypic characteristics, such as its abilities to grow with 15.0% NaCl, hydrolyze urea and reduce nitrate, and its inabilities to grow at 4°C or corrode agar. Thus, based on the phylogenetic, genomic, chemotaxonomic and phenotypic analyses, strain HB14^T^ represents a new species of the genus *Gilvimarinus*, for which the name *Gilvimarinus xylanilyticus* sp. nov. is proposed. Furthermore, the 1,3-xylanases secreted by strain HB14^T^ were found to be 1,3-xylan inducible, and four 1,3-xylanases were identified in the secretome. Among them, Xyn65 is the most abundant and has a novel domain architecture distinct from other 1,3-xylanases. Xyn65 and Xyn80 were biochemically characterized to be two endo-1,3-xylanases. They exhibited the highest activity at neutral pH and mesophilic conditions and released mainly 1,3-xylobiose and 1,3-xylotriose from 1,3-xylan, suggesting that they may have a potential in 1,3-xylooligosaccharides preparation. This is the first report on the identification and characterization of 1,3-xylanases derived from *Gilvimarinus,* which indicates that *Gilvimarinus* may play a role in 1,3-xylan degradation in the ocean.

### Description of *Gilvimarinus xylanilyticus* sp. nov.

*Gilvimarinus xylanilyticus* (xy.la.ni.ly’ti.cus. N.L. neut. adj. *xylanum*, xylan; N.L. masc. adj. *lyticus*, dissolving; from Gr. masc. adj. *lytikos*, dissolving; N.L. masc. adj. *xylanilyticus*, hydrolyzing xylan).

Cells are Gram-stain-negative, aerobic, rod-shaped (approximately 0.3–0.7 μm wide and 1.1–2.7 μm long) and motile with a single polar flagellum. Colonies are circular with regular edges, slightly convex, opaque and approximately 0.5–1.8 mm in diameter on MAC 4 days-incubation at 30°C. Growth occurs at 10–40°C (optimum, 30°C) and pH 6.0–9.0 (optimum, 7.5) with 1.0–15.0% NaCl (optimum, 3.0–5.0%). Cells are positive for oxidase, catalase, 1,3-xylanase, amylase and cellulase, but could not hydrolyze casein and Tween 80. In the API ZYM strips, cells are positive for alkaline phosphatase, esterase (C4), esterase lipase (C8), leucine arylamidase, valine arylamidase, cystine arylamidase, acid phosphatase, naphthol-AS-B1-phosphophydrolase, α-galactosidase, β-galactosidase, α-glucosidase, N-acetyl-β-glucosaminidase, but negative for lipase (C14), trypsin, α-chymotrypsin, β-glucuronidase, β-glucosidase, α-mannosidase and α-fucosidase. In API 20 NE tests, cells are positive for nitrate reduction, urease and aesculin hydrolysis, but negative for indole production, acid production from glucose, arginine dihydrolase, β-galactosidase, gelatine hydrolysis, or assimilation of glucose, arabinose, mannose, mannitol, N-acetyl-glucosamine, maltose, gluconate, caprate, adipate, malate, citrate and phenyl-acetate. On Biolog GEN III microplates (7 days), dextrin, maltose, trehalose, cellobiose, gentiobiose, *α*-D-lactose, melibiose, methyl-β-D-glucoside, D-salicin, *α*-D-glucose, glucuronamide and L-malic acid are oxidized; turanose, raffinose, D-mannose, D-fructose, D-galactose, D-fucose, L-fucose, L-rhamnose, D-glucose-6-phosphate, D-fructose-6-phosphate, L-glutamic acid, L-histidine, D-galacturonic acid, L-galactonic acid lactone, D-gluconic acid, D-glucuronic acid, *α*-ketoglutaric acid, acetoacetic acid and acetic acid are weakly oxidized; sucrose, stachyose, *N*-acetyl-D-glucosamine, *N*-acetyl-β-D-mannosamine, *N*-acetyl-D-galactosamine, *N*-acetyl neuraminic acid, 3-methyl glucose, inosine, D-sorbitol, D-mannitol, D-arabitol, *myo*-inositol, glycerol, D-aspartic acid, D-serine, gelatin, glycyl-L-proline, L-alanine, L-arginine, L-aspartic acid, L-pyroglutamic acid, L-serine, pectin, mucic acid, quinic acid, D-saccharic acid, *p*-hydroxyphenylacetic acid, methyl pyruvate, D-lactic acid methyl ester, L-lactic acid, citric acid, D-malic acid, bromosuccinic acid, Tween 40, γ-aminobutryric acid, α-hydroxybutyric acid, β-hydroxy-D,L-butyric acid, α-ketobutyric acid, propionic acid and formic acid are not oxidized. The predominant cellular fatty acids (>10.0%) are summed feature 3 (contains C_16:1_ *ω*6*c* and/or C_16:1_ *ω*7*c*), summed feature 8 (contains C_18:1_ *ω*7*c* and/or C_18:1_ *ω*6*c*) and C_16:0_. The predominant respiratory quinone is ubiquinone-8 and the main polar lipids are diphosphatidylglycerol, phosphatidylglycerol and phosphatidylethanolamine.

The type strain is HB14^T^ (=CCTCC AB 2022109^T^ = KCTC 92379^T^) isolated from a marine green alga (*Chaetomorpha* sp.) collected from Hainan, China. The genomic G + C content of the type strain (calculated from the draft genome) is 54.0 mol%.

## Data availability statement

The datasets presented in this study can be found in online repositories. The names of the repository/repositories and accession number(s) can be found at: https://www.ncbi.nlm.nih.gov/genbank/, ON521705 https://www.ncbi.nlm.nih.gov/genome/, JAMFTH000000000, and https://www.ebi.ac.uk/pride, PXD035564.

## Author contributions

Y-JZ and FZ designed and directed the research. Y-JZ, H-NS, T-TX, and C-MY performed the experiments. Y-JZ, FZ, Y-QZ, and H-NS wrote the manuscript. D-LZ and YZ helped in data analysis. X-LC and X-YZ revised the manuscript. All authors contributed to the article and approved the submitted version.

## Funding

The work was supported by the National Science Foundation of China (U2006205, 42076151, and 41906195, awarded to X-LC, X-YZ, and YZ, respectively), the Natural Science Foundation of Shandong Province, China (ZR2021QC024, awarded to FZ), and the China Postdoctoral Science Foundation (2021 M691960, awarded to Y-QZ).

## Conflict of interest

The authors declare that the research was conducted in the absence of any commercial or financial relationships that could be construed as a potential conflict of interest.

## Publisher’s note

All claims expressed in this article are solely those of the authors and do not necessarily represent those of their affiliated organizations, or those of the publisher, the editors and the reviewers. Any product that may be evaluated in this article, or claim that may be made by its manufacturer, is not guaranteed or endorsed by the publisher.

## Footnotes

^1^ http://www.cazy.org/

^2^ http://www.ezbiocloud.net/

^3^ http://ggdc.dsmz.de/ggdc.php

^4^ https://www.ezbiocloud.net/tools/ani

^5^ http://enve-omics.ce.gatech.edu/aai/

^6^ https://www.expasy.org/
