# Supplementary material for: Gilvimarinus xylanilyticus sp. nov., a novel 1,3-xylanase-secreting bacterium isolated from a marine green alga
Source: Front Microbiol. 2022 Oct 24;13:1006116. doi: 10.3389/fmicb.2022.1006116 (PMC9638140; doi:10.3389/fmicb.2022.1006116)
Supplement: Supplementary file 1 [file Data_Sheet_1.DOCX]

Supplementary Material

**Supplementary Tables**

**Table S1** Primers used in this study.

| Gene product | Primer | Sequence (5’ to 3’) ^a^ |
| --- | --- | --- |
| Xyn65 | Xyn65-F | AAGAAGGAGATATACATATGGTCAGTGGTACCTTGGTTCC |
|  | Xyn65-R | TGGTGGTGGTGGTGCTCGAGAAGCGTTTTCACAAATGCCT |
| Xyn80 | Xyn80-F | AAGAAGGAGATATACATATGGCGATTGATTCCGGCGCGGT |
|  | Xyn80-R | TGGTGGTGGTGGTGCTCGAGAGGCAAAGTGCAGGCATTGT |

^a^ Sequences identical to that of vector pET22b are underlined.

**Table S2** Cellular fatty acid compositions (%) of strain HB14^T^ and *G. chinensis* CGMCC 1.7008^T^.

| Fatty acid | 1 | 2 |
| --- | --- | --- |
| Saturated |  |  |
| C_10:0_ | 0.4 | 0.6 |
| C_11:0_ | 0.1 | 0.2 |
| C_12:0_ | 6.9 | 10.7 |
| C_13:0_ | 0.2 | 0.2 |
| C_14:0_ | 1.4 | 2.9 |
| C_16:0_ | 11.7 | 16.5 |
| C_17:0_ | 0.3 | 0.7 |
| C_18:0_ | 4.9 | 3.5 |
| Unsaturated |  |  |
| C_14:1_ *ω*5*c* | 0.2 | 0.3 |
| C_17:1_ *ω*8*c* | 0.2 | 0.5 |
| C_18:1_ *ω*9*c* | 0.5 | 0.7 |
| Hydroxy fatty acids |  |  |
| C_10:0_ 2OH | 0.2 | 0.4 |
| C_10:0_ 3OH | 4.8 | 4.5 |
| C_11:0_ 2OH | ND | 0.1 |
| C_11:0_ 3OH | 0.1 | 0.1 |
| C_12:0_ 2OH | 2.9 | 2.7 |
| C_12:0_ 3OH | 3.5 | 3.3 |
| C_12:1_ 3OH | 0.1 | ND |
| Methyl fatty acids |  |  |
| 11–methyl C_18:1_ *ω*7*c* | ND | 0.1 |
| Summed feature* |  |  |
| 2 | 0.1 | ND |
| 3 | 37.2 | 35.3 |
| 5 | 0.1 | 0.1 |
| 7 | ND | 0.8 |
| 8 | 24.2 | 15.7 |

Strains: 1, strain HB14^T^; 2, *G. chinensis* CGMCC 1.7008^T^. All data are from this study. ND, not detected. Summed feature 2 comprises iso-C_16:1_ I and/or C_14:0_ 3OH; Summed feature 3 comprises C_16:1_ *ω*6*c* and/or C_16:1_ *ω*7*c*; Summed feature 5 comprises C_18:2_ *ω*6, 9*c* / ante-C_18:0_; Summed feature 7 comprises un18.846 and/or C_19:1_ *ω*6*c*; Summed feature 8 comprises C_18:1_ *ω*7*c* and/or C_18:1_ *ω*6*c*.

**Supplementary Figures**

*Teredinibacter purpureus* Bs12^T^ (MT416120)

*Teredinibacter franksiae* Bsc2^T^ (MT416121)

*Pseudoteredinibacter isoporae* SW-11^T^ (FJ347760)

*Marinibactrum halimedae* Q-192^T^ (AB900126)

*Agarilytica rhodophyticola* 017^T^ (KR610527)

*Thalassocella blandensis* ISS155^T^ (MH732325)

**Strain** **HB14^T^** (**ON521705**)

*Gilvimarinus chinensis* CGMCC 1.7008^T^ (DQ822530)

‘*Gilvimarinus agarilyticus*’ M5c^T^ (GQ872424)

*Gilvimarinus* *japonicus* 12-2^T^ (LC125208)

*Gilvimarinus* *polysaccharolyticus* YN3^T^ (HM437226)

‘*Porticoccus hydrocarbonoclasticus*’ MCTG13d^T^ (JN088732)

*Porticoccus litoralis* IMCC2115^T^ (EF468719)

*Spongiibacter tropicus* CL-CB221^T^ (EF988653)

*Spongiibacter taiwanensis* SPT1^T^ (KT966463)

*Marinimicrobium agarilyticum* DSM 16975^T^ (AY839870)

*Marinimicrobium locisalis* ISL-43^T^ (EU874388)

*Microbulbifer yueqingensis* CGMCC 1.10658^T^ (GQ262813)

*Microbulbifer elongatus* DSM 6810^T^ (AF500006)

*Limnobacter litoralis* NBRC 105857^T^ (AB682299)

100

100

100

100

100

100

97

99

96

97

99

0.02

**Figure S1** Neighbor-joining phylogenetic tree based on 16S rRNA gene sequences of strain HB14^T^ (in bold) and the type strains of its closely related species. Bootstrap values (>70%) based on 1,000 replicates are shown at the branching points. Bar, 0.02 substitutions per nucleotide position. *Limnobacter litoralis* NBRC 105857^T^ was used as the outgroup.

*Teredinibacter purpureus* Bs12^T^ (MT416120)

*Teredinibacter franksiae* Bsc2^T^ (MT416121)

*Pseudoteredinibacter isoporae* SW-11^T^ (FJ347760)

*Marinibactrum halimedae* Q-192^T^ (AB900126)

*Agarilytica rhodophyticola* 017^T^ (KR610527)

*Thalassocella blandensis* ISS155^T^ (MH732325)

*Spongiibacter tropicus* CL-CB221^T^ (EF988653)

*Spongiibacter taiwanensis* SPT1^T^ (KT966463)

**Strain** **HB14^T^** (**ON521705**)

*Gilvimarinus chinensis* CGMCC 1.7008^T^ (DQ822530)

‘*Gilvimarinus agarilyticus*’ M5c^T^ (GQ872424)

*Gilvimarinus japonicus* 12-2^T^ (LC125208)

*Gilvimarinus polysaccharolyticus* YN3^T^ (HM437226)

‘*Porticoccus hydrocarbonoclasticus’* MCTG13d^T^ (JN088732)

*Porticoccus litoralis* IMCC2115^T^ (EF468719)

*Microbulbifer yueqingensis* CGMCC 1.10658^T^ (GQ262813)

*Microbulbifer elongatus* DSM 6810^T^ (AF500006)

*Marinimicrobium agarilyticum* DSM 16975^T^ (AY839870)

*Marinimicrobium locisalis* ISL-43^T^ (EU874388)

*Limnobacter litoralis* NBRC 105857^T^ (AB682299)

100

96

100

97

96

73

100

99

95

50

**Figure S2** Maximum-parsimony phylogenetic tree based on 16S rRNA gene sequences of strain HB14^T^ (in bold) and the type strains of its closely related species. Bootstrap values (>70%) based on 1,000 replicates are shown at the branching points. *Limnobacter litoralis* NBRC 105857^T^ was used as the outgroup.

**
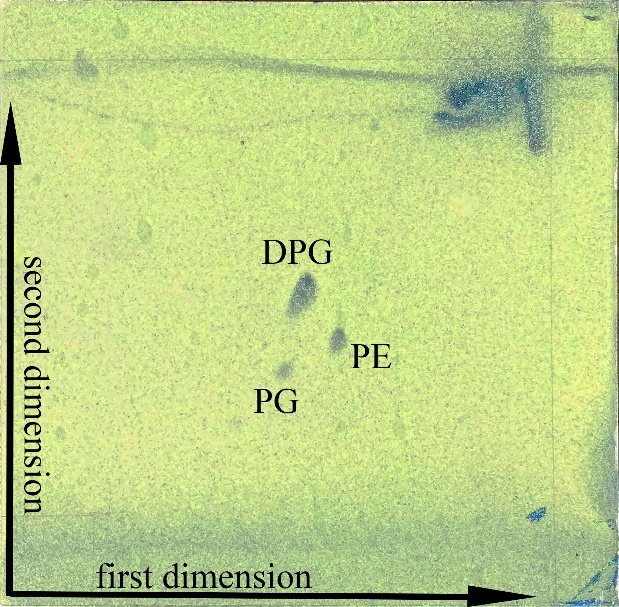
**

**Figure S3** The polar lipids components of strain HB14^T^ stained with ethanolic molybdatophosphoric acid for total lipids. PE, phosphatidylethanolamine; PG, phosphatidylglycerol; DPG, diphosphatidylglycerol.


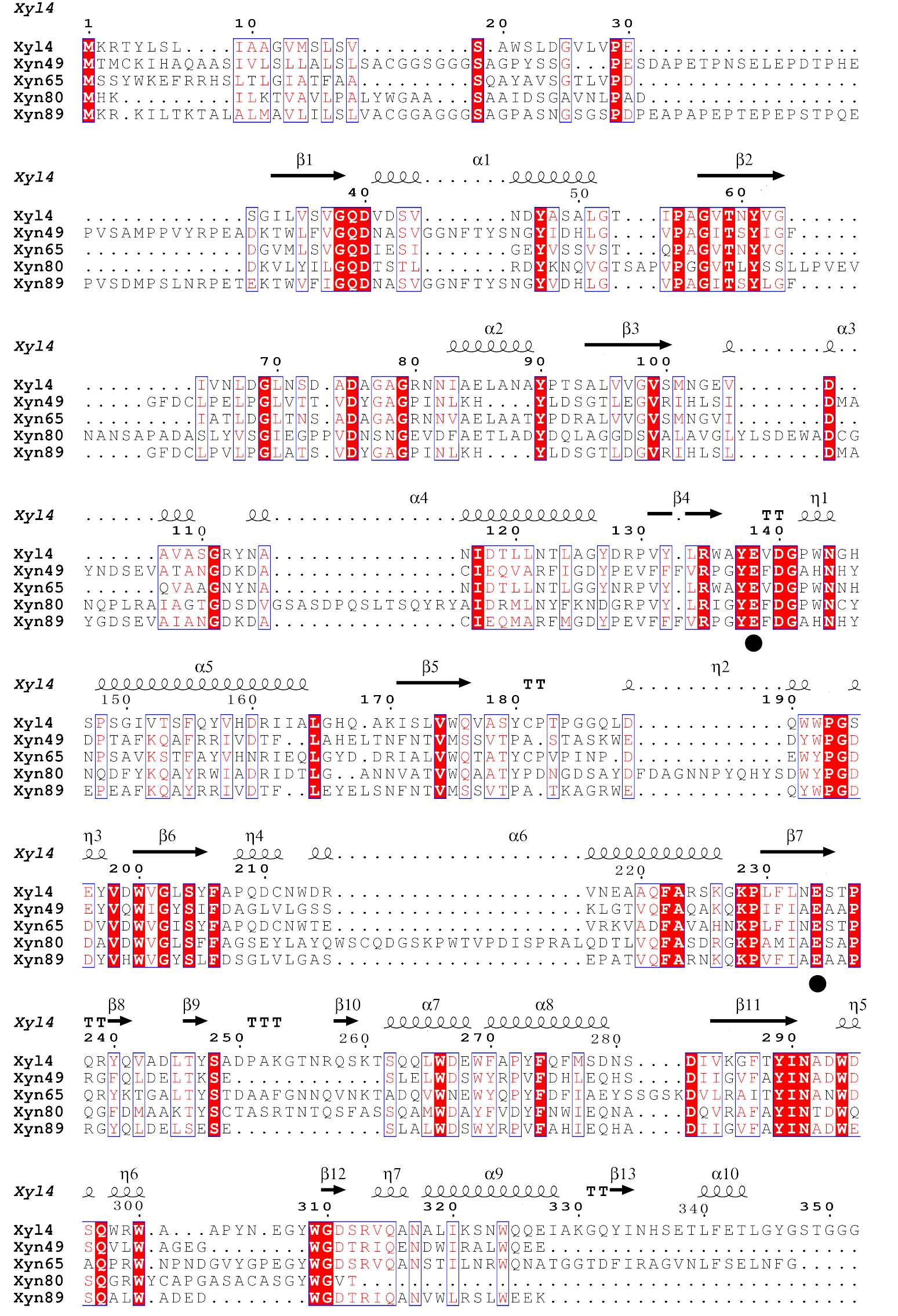


**Figure S4** Multiple sequence alignment of the four 1,3-xylanases secreted by strain HB14^T^ and the previously reported 1,3-xylanases Xyl4 (PDB: 3VPL) in the GH26 family. Black solid circles indicate two catalytic residues in GH26. The secondary structure of Xyl4 is shown above the alignment.
